# A Hybrid Peptide DEFB-TP5 Expressed in Methylotrophic Yeast Neutralizes LPS With Potent Anti-inflammatory Activities

**DOI:** 10.3389/fphar.2020.00461

**Published:** 2020-05-07

**Authors:** Baseer Ahmad, Zhongxuan Li, Quratulain Hanif, Qingyong Hu, Xubiao Wei, Lulu Zhang, Shahzad Akbar Khan, Maierhaba Aihemaiti, Huma Gulzar, Muhammad Shahid, Dayong Si, Rijun Zhang

**Affiliations:** ^1^ State Key Laboratory of Animal Nutrition and Feed Sciences, Laboratory of Feed Biotechnology, College of Animal Science and Technology, China Agricultural University, Beijing, China; ^2^ Computational Biology Laboratory, Agricultural Biotechnology Division, National Institute for Biotechnology and Genetic Engineering, Faisalabad, Pakistan; ^3^ Department of Biotechnology, Pakistan Institute of Engineering and Applied Sciences, Nilore, Islamabad, Pakistan; ^4^ College of Life Sciences, Peking University, Beijing, China; ^5^ Guangzhou Institute of Biomedicine and Health, Chinese Academy of Sciences, Guangzhou, China; ^6^ College of Life Sciences, China Agricultural University, Beijing, China

**Keywords:** expression, hybrid peptide, β-defensins, endotoxin, anti-inflammatory

## Abstract

DEFB-TP5 is a novel auspicious health-beneficial peptide derivative from two naturally occurring peptides, β-Defensin (DEFB) and thymopentin (TP5), and shows strong anti-inflammatory activity and binds to LPS without cytotoxicity and hemolytic effect. Furthermore, the application of DEFB-TP5 peptide is inadequate by its high cost. In the current study, we developed a biocompatible mechanism for expression of the DEFB-TP5 peptide in *Pichia pastoris*. The transgenic strain of hybrid DEFB-TP5 peptide with a molecular weight of 6.7kDa as predictable was obtained. The recombinant DEFB-TP5 peptide was purified by Ni-NTA chromatography, estimated 30.41 mg/L was obtained from the cell culture medium with 98.2% purity. Additionally, The purified DEFB-TP5 peptide significantly (p< 0.05) diminished the release of nitric oxide (NO), TNF-α, IL-6, IL-1β in LPS-stimulated RAW264.7 macrophages in a dose-dependent manner. This study will not only help to understand the molecular mechanism of expression that can potentially be used to develop an anti-endotoxin peptide but also to serve as the basis for the development of antimicrobial and anti-inflammatory agents as well, which also provides a potential source for the production of recombinant bioactive DEFB-TP5 at the industrial level.

## Introduction

Recently, about 500 antipathogenic natural peptides have been revealed to show potential actions against microbes. Segregation of these peptides has been done from an extensive variety of organisms such as vertebrates, invertebrates, bacteria, plants and fungi (Hancock and Chapple, 1999; Shah et al., 2017). Antimicrobial peptides (AMPs) were reflected in one of the exceptional preferences to use as a substitute or in combination with conventional therapeutics. The limited number of antimicrobial, anti-endotoxin, immunomodulatory and anti-inflammatory agents have prompted and reinforced the urgent need to search and identify new compounds. These therapeutic agents’ origin-based antimicrobial peptides work by a novel mechanism of action (Weinstein, 1998; Jones, 2001). Antimicrobial peptides (AMPs) are small in size and that are part of the innate immune system (Zasloff, 2002; Martin et al., 2015; Brandenburg et al., 2016). AMPs can moderate the host immune response including the conscription of immune cells to the site of infection (Hilchie et al., 2013). The immunomodulatory, and anti-inflammatory properties of AMPs can be exploited to treat inflammation and sepsis (Hu et al., 2014; Martin et al., 2015; Matzneller et al., 2017).

β-defensins (DEFB) are the most important components of the immune system and constitute an innate immune defense against an array of enveloped viruses, fungi and bacteria (Weinstein, 1998; Jones, 2001; Eliopoulos et al., 2003; Yi et al., 2014). β-defensins also play various functional roles apart from these common features, including immunomodulatory and chemotaxis effects to immune cells (García et al., 2001; Niyonsaba et al., 2002; Brogden et al., 2003). These outcomes in the obstruction and perforation of the bacterial membrane, cytoplasmic leakage of organelles and bacterial cell death (Gough et al., 1996; Hancock and Chapple, 1999). β-defensins may be acting as ideal candidates for the manufacturing of antibiotics because of their broad microorganism-killing spectrum. β-defensins, function as chemotactic agents for activated neutrophils, macrophages, immature dendritic cells, monocytes, and memory helper T cells (VanderMeer et al., 1995; Scott et al., 2000; Bhattacharjya, 2010), as they impart connection between the innate and adaptive immune system by providing an immunologic response to microbial infection.

Cationic AMPs are evolutionary antique components that impart a role in the innate immune system by blocking several of the actions of LPS (Hancock and Lehrer, 1998). Synthetic and naturally occurring AMPs have a potential ability to lyse bacteria, bind the LPS and reduce the production of nitric oxide (NO), IL-1β, IL-6, TNF-α and other inflammatory mediators (Gough et al., 1996). Unforeseen, according to previous observations, DEFBs have reasonably low LPS-neutralization potency (Scott et al., 2000; Bhattacharjya, 2010; Semple et al., 2010). Therefore, there is a desire need to hybridize DEFB peptide along with immunomodulatory peptide TP5 to enhance the efficacy. The biological activity to thymopentin is reproduced by a synthetic pentapeptide thymopentin (TP-5) whose amino acid sequence corresponds to the region 32-36 of the native hormone (Arg-Lys-Asp-Val-Tyr) (Goldstein et al., 1979). TP5 has been successfully used in humans for the treatment of Immunological parameters in neoplasmic, immune deficiency and autoimmune diseases (Goldstein et al., 1979; Singh et al., 1998). Furthermore, TP5 also contains a particular value in humans with certain recurrent viral diseases (Sundal and Bertelletti, 1994; Fan et al., 2006).

However, the high expense of peptide construction limits its synthesis. The development of peptide antibiotics is challenging to formulate on a scalable and cost-effective method to produce active commercially based products. The application of recombinant heterologous expression methods for peptides is a solution to this problem. The heterologous protein expression system methylotrophic *Pichia pastoris* (*P. pastoris*) has been used extensively (Cereghino and Cregg, 2000). *P. pastoris* is economical for large scale expression its comprises of alcohol oxidase-1 (AOX1) gene promoter which is repressed by glucose and glycerol and induced by methanol (Sreekrishna et al., 1997).

However, to date, there have been no studies in which the DEFB-TP5 peptide has been expressed in the *P. pastoris* system. In the present study, we assumed that the combination of DEFB (39 amino acid) and TP5 (5 amino acid) may have amplified LPS neutralization, inhibit the growth of Gram-negative bacteria, anti-inflammatory action along with minimum cytotoxic properties. Consequently, we incorporated and expressed the hybrid peptide DEFB-TP5, in the yeast expression system and explored its activities.

## Materials and Methods

### Materials

#### Strains, Vectors, and Reagents

The expression and cloning plasmid pPICZαA, strain *E.coli* DH5α, strain *Pichia pastoris* X-33 and Zeocin™ were bought form (Invitrogen, Carlsbad, CA, USA). The restriction enzyme *EcoR I, Not I, Sac I* (TaKaRa Biotechnology, Dalian, China) and PCR reagents, DNA Marker (50 and 100 bp) were purchased from Tiangen Biotech (Beijing, China). The *E.coli* LPS (O55: B5) was obtained from Sigma (USA). The Gel Extraction kit, Plasmid Mini kit, Yeast DNA extraction kit, and Protein markers (Sangon Biotech, Shanghai, China) were regularly used in our research laboratory.

### Construction of Recombinant Expression Plasmid pPICZαA-DEFB-TP5

The preferred codons of *P.pastoris* based on the novel DEFB-TP5 peptide amino acid sequence were selected and optimized *via* JAVA codon adaptation tool (JCAT) http://www.jcat.de/Start.jsp). The two oligonucleotides (184 bp) analogous to the partial sequence (sense and antisense) strands of the DNA sequence were synthesized. A restriction site was allowed for the expression of native N-terminus of DEFB-TP5 and introduced in-frame cloning into the α-factor secretion signal of the pPICZα-A expression vector. At the C-terminus stop codon and *Not I* restriction site was placed with 6×His-tagged. The full-length DNA template (DEFB-TP5) was procured by using primers (P1. (5′ CGCGGATCCAACTGGTACGTTAAGA-3′; P2. 5′ TCCCCCGGGTCAATGATGATGATG-3′) and PCR (35 cycles 94 ˚C for the 5min; 94 ˚C for the 30s; 55 ˚C for 30s, 72 ˚C for 50 s) and a final cycle at 72 ˚C for 10 min. The PCR product which encodes DEFB-TP5 peptide was digested with *EcoR I* and *Not I* enzymes and ligated into the *EcoR I/Not I*-digested pPICZα-A. This recombinant expression vector (pPICZαA-DEFB-TP5) was transformed into competent *E. coli* DH5α and confirmed by sequencing.

### Selection and Transformation of pPICZαA-DEFB-TP5 Into *P. pastoris*

The *P. pastoris* X-33 cells were metamorphosed and expression plasmid was linearized earlier by *Sac I* followed electroporation manufacturer’s instructions. An alone pPICZα-A vector was also inserted into *P. pastoris* X-33 cells which represented as a negative control. After transformation, allow to grow zeocin-resistant colonies in YPDS medium (1% yeast extract, 2% peptone, 2% dextrose, 1 M sorbitol,2% agar, and 100 µg/ml Zeocin). Later, PCR and sequencing were used to define the DEFB-TP5 coding sequence insertion in the genome of host cells by a screening of the resistant colonies.

### Expression of Recombinant Hybrid DEFB-TP5 Peptide Into *P. pastoris*

The recombinant hybrid DEFB-TP5 was expressed by applying the optimal condition (0.5% methanol v/v, pH 5.5, and temperature 28 ˚C) in Buffered Methanol-Complex medium(BMMY). The positively transformed yeast cells were cultured for about 20 h in a shaking flask comprising 50ml Buffered Glycerol Complex Medium (BMGY, 1% yeast extract,2% peptone, 100mM potassium phosphate buffer, pH 5.0, 1.34% YNB, 4 × 10^-5^% biotin, and 1% glycerol) to OD_600_ = 4.0. Cells were garnered by centrifugation at 2000 × g for 8 min at room temperature and resuspended to an OD_600_ of 1.0 in BMMY medium (1% yeast extract, 2% peptone, 100mM potassium phosphate buffer, pH 5.0, 1.34% YNB, 4 ×10^-5^% biotin, and 0.5% methanol) to induce expression of the recombinant peptide. After 144 h methanol induction, 50µL expression medium was proceeded and analyzed by Tricine-sodium dodecyl sulfate-polyacrylamide gel electrophoresis (Tricine-SDS-PAGE). The concentrations were determined through Bradford method by using bovine serum albumin as standard (Bradford protein assay kit, Sangon Biotech, Shanghai, China (Ausubel et al., 1999).

### Purification of Recombinant Hybrid DEFB-TP5 Peptide

Purification of the recombinant hybrid peptide was done by Ni-NTA column method with slight modification as previously described (Wei et al., 2018). The expressed culture medium centrifuged (12,000 × g for 20 min at 4 ˚C) and the supernatant was collected. The 0.45mm filter membrane was used to filter the collected supernatant and dialyzed several times with 3 volumes of binding buffer (20mMNaH2PO4, 300mMNaCl, 10mM imidazole, pH 7.4) to eliminate the medium components. The column was equilibrated with a binding buffer that was earlier charged with NiCl2 and then filtered supernatant applied to Ni-NTA column overnight at 4 ˚C. Furthermore,with the help of washing buffer 1 (20mMNa H2PO4, 300mMNaCl, 20mMNaCl, 20mMimidazole, pH 7.4) and washing buffer 2 (20mMNaH2PO4, 300mMNaCl, 60mM imidazole, pH 7.4), column was rinsed successively. The certain peptide was eluted with 1 ml elution buffer (20m NaH2PO4, 300 mM NaCl, 400 mM imidazole and 500 mM imidazole with pH 7.4) for five times. Tricine-SDS-PAGE, silver staining, and Bandscan 5.0 software were used to analyze the eluted fractions.

### Efficacy of Hybrid DEFB-TP5 Peptide on Gram-Negative Bacteria

The antimicrobial activity of DEFB-TP5 was tested against *E.coli* C 84002 by the agar diffusion method. The indicator strain dilution was spread on Mueller-Hinton broth (MHB) plates. Cylinders were located on the agar surface and 100 μL of purified recombinant DEFB-TP5 and D-PBS was added to each cylinder. Ampicillin (100 U) was used as a positive control and the inhibition zone was measured after overnight incubation at 37°C.

### Determination of Lipopolysaccharide (LPS) Neutralization

Chromogenic Limulus amebocyte lysate (LAL) assay was used to evaluate the neutralization of LPS by the parental (TP5) and hybrid (DEFB-TP5) peptide. LPS (1EU/ml) and different concentrations of peptides (0 to 60µg/ml) were incubated at 37°C. To the LAL reagent, an equal volume of 50 µL aliquots of the mixture was added, and then the resulted mixtures were incubated for 10 min at 37°C. Upon the addition of 100µL of a chromogenic substrate solution, the development of yellow color appears. HCl was then added in order to stop the reaction and absorption was measured at 545 nm (Kim et al., 2011; Ahmad et al., 2019).

### Hemolytic Activity

The hemolytic activity of TP5 and DEFB-TP5 was indomitable by using heparinized mouse red blood cells (RBCs) as described earlier (Shahid et al., 2017; Ahmad et al., 2019). The 4mL fresh mouse RBCs were centrifuged (1500 rpm for 10 min at 4°C) and washed three times with diluted 10% hematocrit. The recombinant DEFB-TP5 peptide was dissolved in phosphate-buffered saline (PBS) with various concentrations (30 to 60µL) and incubated for 1 h at 37°C. The sample was centrifuged (at 3500 rpm for 5 min) and absorbance (Abs) of the supernatant was measured at 414 nm.

$$\text{\% hemolysis}=\frac{\text{Abs }414\text{ of a sample}-\text{Abs }414\text{ of negative control }\left(\text{PBS}\right)\times 100}{\text{Abs }414\text{ of positive control }\left(\text{Triton}-\text{X}100\right)}$$

### Cell Culture

The mouse macrophage (RAW264.7) cells were cultivated in Dulbecco’s Modified Eagle Medium (DMEM) to amplify with antibiotics (100 µg/ml streptomycin and 100U/ml penicillin) and 10% fetal calf serum under an atmosphere containing 5% CO2 in a humified chamber.

### Lactate Dehydrogenase Activity (LDH) Assay

In order to evaluate the cytotoxic influence of LPS, TP5 and DEFB-TP5 on RAW 264.7, macrophage LDH kit (Dojingdo Laboratories, Kumamoto, Japan) assay were used. The cells (1 × 105 cells/mL) were infected by LPS alone (1 µg/ml), TP5+LPS and DEFB-TP5 + LPS (30 to 60 µg/ml) for about 24 h. After incubation, the supernatants were collected and analyzed according to manufacturers’ instructions (Ahmad et al., 2019).

### Inhibition of Nitric Oxide (NO) Production in LPS-Stimulated RAW264.7 Macrophages

The murine RAW264.7 cells were incubated with LPS only (1 µg/ml) and LPS plus the different concentrations of parental and hybrid peptides (30 to 60 µg/ml). NO production was determined by the collected supernatant. A 100 µL aliquot of the culture medium was mixed with the same volume of Griess reagent (1%sulfanilamide in 5% phosphoric acid and 0.1% naphthylethylene diamine dihydrochloride) and further incubated for 15 min (Green et al., 1982). The secretion of NO was measured at 550 nm by using an enzyme-linked immunosorbent assay (ELISA) reader.

### Evaluation of Pro-inflammatory Cytokines in LPS-Induced Murine RAW264.7 Macrophages

After the infection of LPS (1µg/ml) to RAW 264.7 cells (5x105/well), in the presence or absence of TP5 and hybrid DEFB-TP5 peptide (30 to 60µg/ml). The expression level of a proinflammatory cytokine TNF-α, IL-1β and IL-6 were accessed by using ELISA kit (could-clone corp, Houston, USA). The levels were quantified at 450 nm absorbance.

### Statistical Analyses

All the data were presented as mean ± S.D. For statistical analysis, one-way analysis of variance (ANOVA) and Duncan’s multiple range tests were used and carried out with SPSS 19.0 (SPSS Inc., Chicago, IL, USA). Differences with a *P* < 0.05 and *P* < 0.01 were considered statistically significant.

## Results

### Construction of Expression Recombinant Plasmid pPICZαA-DEFB-TP5

The recombinant DEFB-TP5 gene was amplified by PCR, it was tagged with 6 × Histadine at C-terminal that facilitate the upcoming peptide purification. At 5′and 3′end, the restriction enzyme *EcoR I* and *Not I* was attached. The DEFB-TP5 peptide was synthesized and inserted into pUC57vector after double digested with *EcoR I* and *Not I*. This fragment was cloned in the frame of the α-factor secretion signal, downstream of the AOX1 promoter of the *P. pastoris* expression plasmid pPICZαA to outcome in expression vector named pPICZαA-DEFB-TP5. The correction of the insertion was then confirmed by PCR and direct nucleotide sequencing (data not shown). The construction process of pPICZαA-DEFB-TP5 as shown in (**Supplementary Figure 1**).

### Expression and Purification of Hybrid DEFB-TP5 Peptide

**Figure 1:**
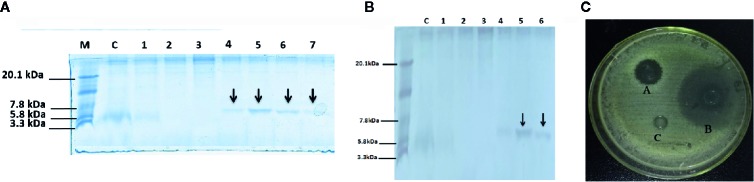
Tricine-SDS-PAGE and analysis of recombinant peptide, **(A)** Tricine-SDS-PAGE of the cell culture media from *P.pastoris* expressing secreted DEFB-TP5. Lane M, mass weight markers; Lane C, control (blank PpICZαA and X-33 strain); Lane 1 to 7 (supernatant X33/PpICZαA-DEFB-TP5) peptide expression after methanol (12 to 144 h) induction and arrow in the lane indicated 6.7 kDa peptide **(B)** Tricine-SDS-PAGE of Purified secreted recombinant hybrid peptide DEFB-TP5. Lane M, mass weight markers; Lane C, control (blank PpICZαA and X-33 strain); Lane 1-6 purified X33/DEFB-TP5 extract with different concentrations of imidazole and arrow in the lane indicated 6.7 kDa (400 and 500mm imidazole) polypeptide. **(C)** The antimicrobial activity of recombinant DEFB-TP5 against *E.coli* C 84002, A: 100 U Ampicillin sodium, B: recombinant hybrid DEFB-TP5 peptide (concentration 5mg/L), C: The negative control, sodium phosphate buffer (PBS).

The expression plasmid pPICZαA-DEFB-TP5 was linearized with *Sac I* and transferred to *P. pastoris* X-33 by electroporation. Ninety-two Zeocin-resistant transformants were screened through colony-PCR. Our results revealed that all positive transformants had the target DEFB-TP5 sequence and successfully incorporated into the host cells. These recombinant DEFB-TP5 positive colonies were induced by adding 0.5% pure methanol to express peptide for a continuous six days. The recombinant peptide started to be detected at 24 h post-induction and the signal peptide had been removed from the N-terminus and secreted into the culture medium (**Figure 1A**). The hybrid DEFB-TP5 peptide was purified by the NI-NTA chromatography column. The pure recombinant hybrid peptide was eluted with 400mM and 500 mM imidazole which seemed like a single band with a molecular weight of approximately 6.7 kDa as expected on SDS-PAGE followed by silver staining as presented in (**Figure 1B**). The yield of purified peptide was approximately 30.41mg/L assessed by the BSA method (**Supplementary Figure 2**). The 200 ml pure peptide was further subjected to RP-HPLC to determined the purity of the hybrid peptide. Our results revealed 98.2% purity and the eluted peaks retention time was 12.631 min (**Supplementary Figure 3**). Furthermore, the recombinant DEFB-TP5 peptide sequence identified by LC-MS (**Supplementary Figure 4**).

### Antimicrobial Susceptibility Towards Gram-Negative Bacteria

The hybrid DEFB-TP5 has been evaluated against common bacterial pathogens by using the agar well diffusion method. The purified hybrid DEFB-TP5 peptide (5 mg/mL) revealed high efficacy against *E.coli* C84002 as compared with ampicillin and control. These results provide evidence that recombinant hybrid DEFB-TP5 introverted the growth of Gram-negative bacteria (**Figure 1C**).

### Neutralization of LPS

**Figure 2:**
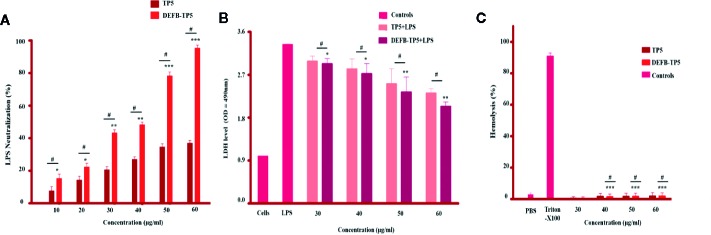
LPS neutralization, cytotoxicity, and hemolytic activity of parental and recombinant DEFB-TP5 peptide. **(A)** Endotoxin binding by means of an endotoxin quantitation kit. Mean values presented; n = 3 ± SD (*p < 0.05, **p < 0.01 and ***p < 0.001 showed comparison of LPS vs. DEFB-TP5. Whereas, ^#^p < 0.05 showed significant difference compared with parental TP5 peptide). **(B)** hybrid peptide reduced LDH in the supernatant of LPS-stimulated mouse RAW264.7 macrophages. Data represented as mean ± standard deviation (SD). While, *p < 0.05 and **p < 0.01 vs. LPS and ^#^p < 0.05 indicates significant difference compared with parental TP5 peptide. **(C)** Hemolytic effect of DEFB-TP5 in contradiction of mouse RBCs. The data resemble the mean values of 3-independent experiments and the (% age) of hemolysis ± standard deviation (***p < 0.001 vs. Triton X-100) While, ^#^p < 0.05 showed a comparison with TP5.

We predicted that the recombinant hybrid DEFB-TP5 bind LPS because under a physical situation it has a positive net charge. The Chromogenic End-point Tachy plus Amebocyte Lysate (CE TAL) assay is an immense indicator of the existence of free non- neutralized endotoxin. We investigated the ability of hybrid peptide to neutralize LPS by using this assay. Our result demonstrated that TP5 (50 and 60 µg/ml) was adept at neutralizing LPS (34.66% ± 0.471, 37.20% ± 0.816% respectively) and DEFB-TP5 (78.23% ± 3.125 and 98.95% ± 4.136 respectively) in a dose-dependent manner (**Figure 2A**). Comparatively, hybrid DEFB-TP5 peptide significantly (p < 0.05) increased the neutralization of LPS than a parental peptide.

### Cytotoxicity and Hemolytic Activity

A possible constraint to the development of the recombinant hybrid DEFB-TP5 peptide as antibiotics is their potential to cause cytotoxicity and damage the mammalian cells. To assess this limitation, we examined their cytotoxic and hemolytic capability towards LPS-infected murine RAW264 macrophages and lyse mouse erythrocytes. Our cytotoxicity assay exposed that only the LPS-infected group released a significantly higher level of LDH (3.14 ± 0.071) at 24h as compared with the collective treatment of LPS and DEFB-TP5 peptide (30 to 60 µg/ml), and control group. This result specifies that LPS seriously damaged the murine RAW264.7 macrophages but various concentrations of hybrid DEFB-TP5 peptide significantly neutralized the LPS and reduced the LDH level (2.33 ± 0.065) at 50µg/ml, (2.03 ± 0.045) at 60µg/ml respectively (**Figure 2B**). Moreover, the hybrid peptide reduced LPS-induced cytotoxicity more than parental peptide.

In case of hemolysis, the hybrid peptide treated cells perceived significantly (p < 0.001) 0% hemolysis as compared with the control group (**Figure 2C**). Notably, these outcomes provide evidence that parental and hybrid DEFB-TP5 peptide doesn’t have broadly cytotoxic and hemolytic properties.

### Inhibition of NO and Inflammatory Cytokines Production in LPS-Stimulated Murine RAW264.7 Macrophages

**Figure 3:**
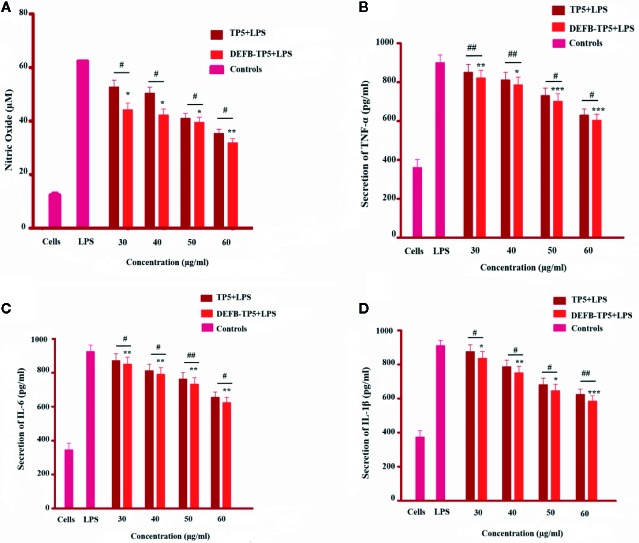
Effect of TP5 and recombinant DEFB-TP5 peptide on LPS-infected inflammatory response in mouse RAW264.7 macrophages. **(A)** Nitric oxide (NO) production, **(B)** level of Tumor necrosis factor-a, **(C)** Interleukin-6, and **(D)** Interleukin-1b. Standards are means ± SD of three independent experiments. *p < 0.05, **p < 0.01, ***p < 0.001, showed comparsion with LPS. While, #p < 0.05 and ##p < 0.01 indicates significant difference compared with parental TP5 peptide.

To further investigate the anti-inflammatory activity of DEFB-TP5 peptide, we measured the NO production in LPS-infected RAW264.7 macrophages. As shown in **Figure 3A** the recombinant hybrid peptide significantly (p< 0.05) inhibited NO production (35 µM at 50 µg/ml and 26 µM at 60 µg/ml) in a dose-dependent manner in LPS-stimulated RAW264.7 cells. Furthermore, to identify the anti-inflammatory activities of hybrid DEFB-TP5 peptide, we measured the capability to diminish proinflammatory cytokines production in LPS-induced RAW264.7 macrophages. Cells were treated with recombinant DEFB-TP5 peptide at (30 to 60 µg/ml) and TNF-α, IL-6, and IL-1β were measured and compared with the controls group. However, the recombinant hybrid DEFB-TP5 peptide exhibited most proficiently inhibition of TNF-α (701 pg/ml at 50 µg/ml and 603 pg/ml at 60 µg/ml respectively) shown in (**Figure 3B**). A similar pattern was observed in case of IL-6 and IL-1β that the only LPS-infected cell as produced a high level of cytokines but the DEFB-TP5 decreased the level (732 pg/ml at 50 µg/ml and 623 pg/ml at 60 µg/ml respectively) (**Figure 3C**) and (645 pg/ml at 50 µg/ml and 584 pg/ml at 60 µg/ml respectively) presented in (**Figure 3D**). Our result implying that recombinant hybrid DEFB-TP5 peptide is a potent anti-inflammatory agent. Moreover, the hybrid peptide DEFB-TP5 exhibited more anti-inflammatory activities as compared to parental peptide TP5 (**Figures 3A–D**).

## Discussion

In current years, the researchers have tried to alter the amino acid sequence of the parental peptides to improve their expression and efficacy. However, the specific part of the amino acid sequence of the peptide has an inordinate influence on the antibacterial and anti-inflammatory activities. The appropriate replacement of the conserved sequence doesn’t affect its activity but some suitable substitutions enhance the efficiency of hybrid peptides (Shah et al., 2017). Hybridization of various parental peptides and alterations in physicochemical properties (net charge, α-helix structure, and hydrophobicity) by reducing the size are the common practices for the development of potent hybrid peptides (Brown et al., 2008).To date, there are different systems for the manufacturing of peptides such as extraction from natural sources, chemical, synthesis, and DNA recombinant technology (Van Harten et al., 2018). Among all of them, the methylotrophic yeast expression is an economical system for the construction of recombinant fusion proteins (Jiménez et al., 2015).

In the present study, hybrid peptide DEFB-TP5 was successfully expressed in *P. pastoris*. Comparatively, two main points differentiate the yeast expression from bacterial expression. Firstly, the yeast expression vector pPICZαA comprises an alcohol oxidase gene (AOX1) promoter and an α-factors signal peptide has ability to stably integrate expression plasmids at specific sites and secrete heterologous proteins. Secondly, the *P.pastoris* system operates and promotes disulfide bonding which would be important for the activation of disulfide present in DEFD-TP5 recombinant peptide expressed and secreted into the medium. After expression, purification was facilitated by a poly-histidine tag that enables separation of soluble, secreted DEFB-TP5 from host strain fermentation supernatant by Ni^2+^ affinity chromatography. As expected, the target peptide DEFB-TP5 6.7 kDa was detected on SDS-page and its concentration was 30.41 mg/L. The peptide yield is greater than earlier described such as T-catesbeianin-1 (Xu et al., 2018), ceropinAD (Jin et al., 2009), and CA-MA (Xu et al., 2007).

In the current study, purified DEFB-TP5 peptide was further tested for antimicrobial, LPS neutralization, cytotoxicity, and hemolytic activity. These features of the recombinant peptide are to be used as beneficial therapeutics. LPS comprises of three parts i. Lipid A, ii. O antigen iii. Polysaccharide core. Lipid A is a toxically active part that caused fever, septic shock, and leukocytosis (Rapicavoli et al., 2018). Lipid A is also accountable for the stimulation of LAL reagent (Iwanaga et al., 1992). In the present study, the LAL assay revealed that recombinant DEFB-TP5 peptide neutralizes endotoxin by binding lipid A. Higher LPS neutralization activity of hybrid DEFB-TP5 peptide was observed than that of CA-MA peptide which was reported previously (Lee et al., 2016). Additionally, the parental antimicrobial peptides have effective activities against microbes but also exhibit cytotoxic and hemolytic effects toward mammalian cells (Zasloff, 1987).

The net charge (+7 to +9) and amphipathicity of the AMPs permit strong antibacterial and anti-inflammatory activity (Zhang et al., 2016). Our peptide comprises the N-terminal region with polar β- Defensin amino acid and a C-terminal region with TP5 amino acid. This combination increases the net charge to +8 and hydrophobicity and supported our hypothesis and previous studies (Gutsmann et al., 2010; Kaconis et al., 2011; Heinbockel et al., 2013; Ahmad et al., 2019). These features presumed to imitate robustly electrostatic interaction between hybrid DEFB-TP5 peptide and LPS. The recombinant hybrid DEFB-TP5 peptide showed more potent antibacterial activity against *E.coli* C84002 comparable to that of ampicillin. However, hybrid DEFB-TP5 peptide exhibited neglectable cytotoxicity and hemolysis.

During multiplication or lysis of bacteria superficial certainly secreted endotoxin (Walters et al., 2010). We further evaluated LPS-infected production of NO and pro-inflammatory cytokines TNF-α, IL-6, IL-1β in mouse RAW264.7 macrophages. The immune activated macrophages secrete NO at the site of inflammation to heal impairment and eradicate the cause (Koh and DiPietro, 2011). However, excessive secretion of NO leads to inflammation. Therefore, plummeting the production of NO could be a new tactic against inflammatory disorders. Inflammation is convoluted chronic diseases i.e cardiovascular and cancer (Moutsopoulos and Madianos, 2006). Endotoxin is a major constituent of the outer membrane of gram-negative bacteria and can promote proinflammatory cytokines in phagocytic cells (Chen et al., 2019). Consequently, diminishing proinflammatory response is imperative to reduce inflammatory disease. Comparatively, the recombinant DEFB-TP5 peptide more efficiently inhibited the production of cytokines that previously identified peptides such as SPHF1 (Ahn et al., 2012) and lunasin-4 (Zhu et al., 2018). Overall, these interpretations designate that recombinant hybrid DEFB-TP5 is a promising peptide that might be industrialized.

## Conclusions

For the first time, we reported a successful expression method for the hybrid DEFB-TP5 peptide in *P.pastoris* with expression vector PpICZαA. To achieve a higher expression Highly active recombinant peptide DEFB-TP5 with molecular weight 6.7kDa was obtained. DEFB-TP5 potently neutralizes LPS with no cytotoxic and hemolytic activity. Additionally, DEFB-TP5 novel peptide exhibited antimicrobial and anti-inflammatory activity by inhibiting cytokine formulation, including NO, TNF-α, IL-6, IL1β. This research study delivered a probable strategy for assembly of bioactive DEFB-TP5 in the industry and might be helpful for binding endotoxin and preventing inflammatory diseases.

## Data Availability Statement

All datasets generated for this study are included in the article/**Supplementary Material**.

## Author Contributions

Wrote the manuscript: BA and ZL. Accomplished the trials: BA, QH, ZL, and QHu. Conceived and planned experiments: BA, QH, and RZ. Analyzed the data: BA, QH. Review and English proficiency: BA, QH, MS. English grammar check: LZ, XW, SK, MA, and HG. Guided and supported the experiments: DS and RZ. Supervision: RZ. All authors abetted to read and sanctioned the final article.

## Funding

This research project was sponsored by the National Key Research and Development Program of China (No. 2018YFD0500600), National Natural Science Foundation of China (No. 31272476 and 31572442), and the Specific Research Grant for the Doctoral Program of Higher Education of China (No. 20110008110002).

## Conflict of Interest

The authors declare that the research was conducted in the absence of any commercial or financial relationships that could be construed as a potential conflict of interest.

## Supplementary Material

Supplementary Figure 1 Construction of recombinant expression vector pPICZαA-DEFB-TP5.

Supplementary Figure 2 Protien concentration estimated by bradford standard curve with formula y = 0.0026x + 0.3673 R^2^ = 0.9947.

Supplementary Figure 3 RP-HPLC RP-HPLC elution profile of DEFB-TP5. Determination showed that the purity of DEFB-TP5 was 98.2%.

Supplementary Figure 4 Liquid Chromatography-Mass Spectrometry (LC-MS) analysis of recombinant purified hybrid DEFB-TP5 peptide.
