# Supplementary figures and images for: A Hybrid Peptide DEFB-TP5 Expressed in Methylotrophic Yeast Neutralizes LPS With Potent Anti-inflammatory Activities
Source: Front Pharmacol. 2020 May 7;11:461. doi: 10.3389/fphar.2020.00461 (PMC7221121; doi:10.3389/fphar.2020.00461)

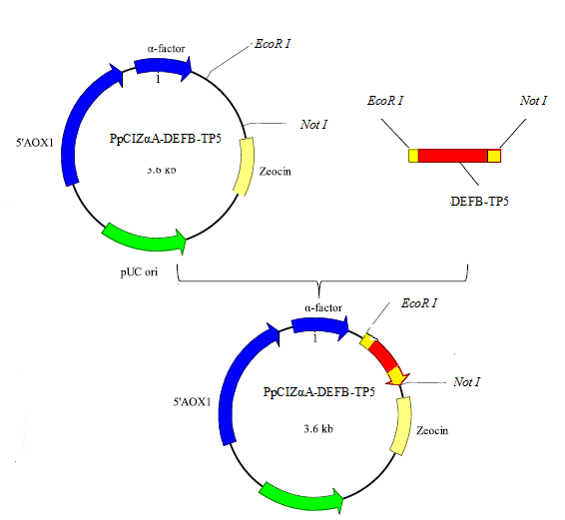

Supplement: Supplementary Figure 1 — Construction of recombinant expression vector pPICZαA-DEFB-TP5. [file Image_1.tif]

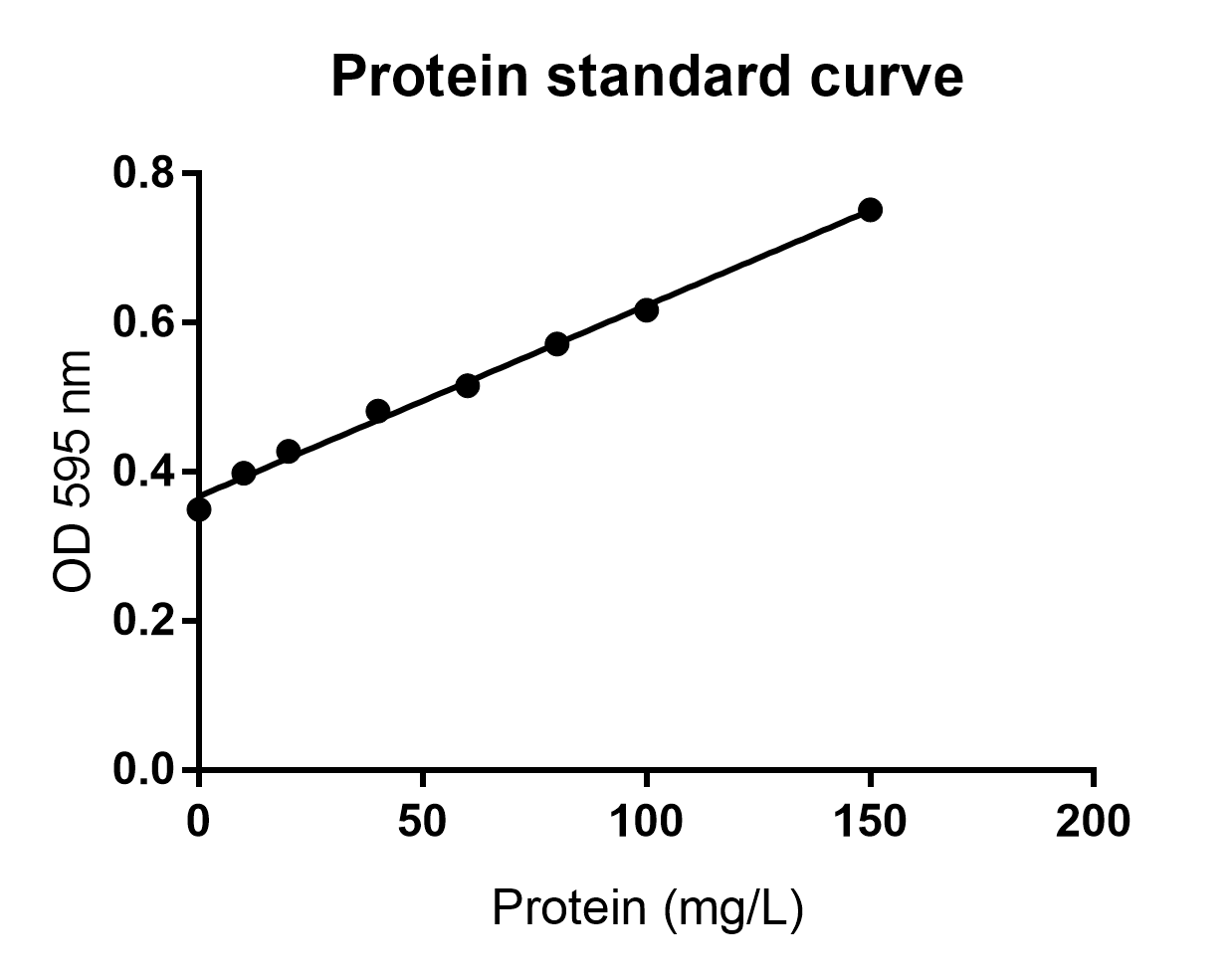

Supplement: Supplementary Figure 2 — Protien concentration estimated by bradford standard curve with formula y = 0.0026x + 0.3673 R2 = 0.9947. [file Image_2.tif]

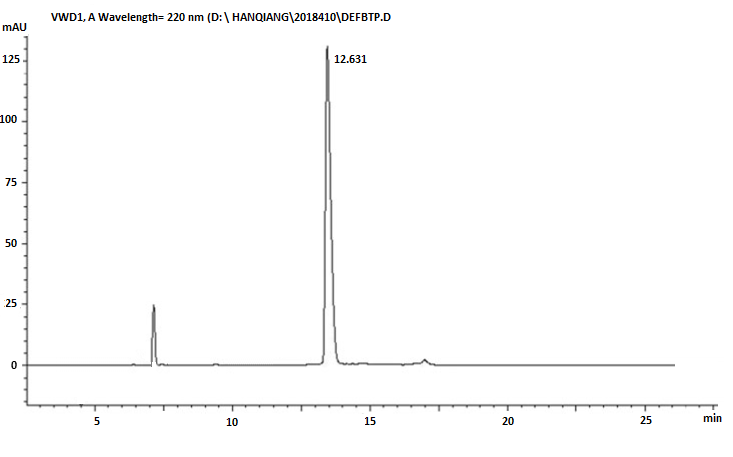

Supplement: Supplementary Figure 3 — RP-HPLC RP-HPLC elution profile of DEFB-TP5. Determination showed that the purity of DEFB-TP5 was 98.2%. [file Image_3.tif]

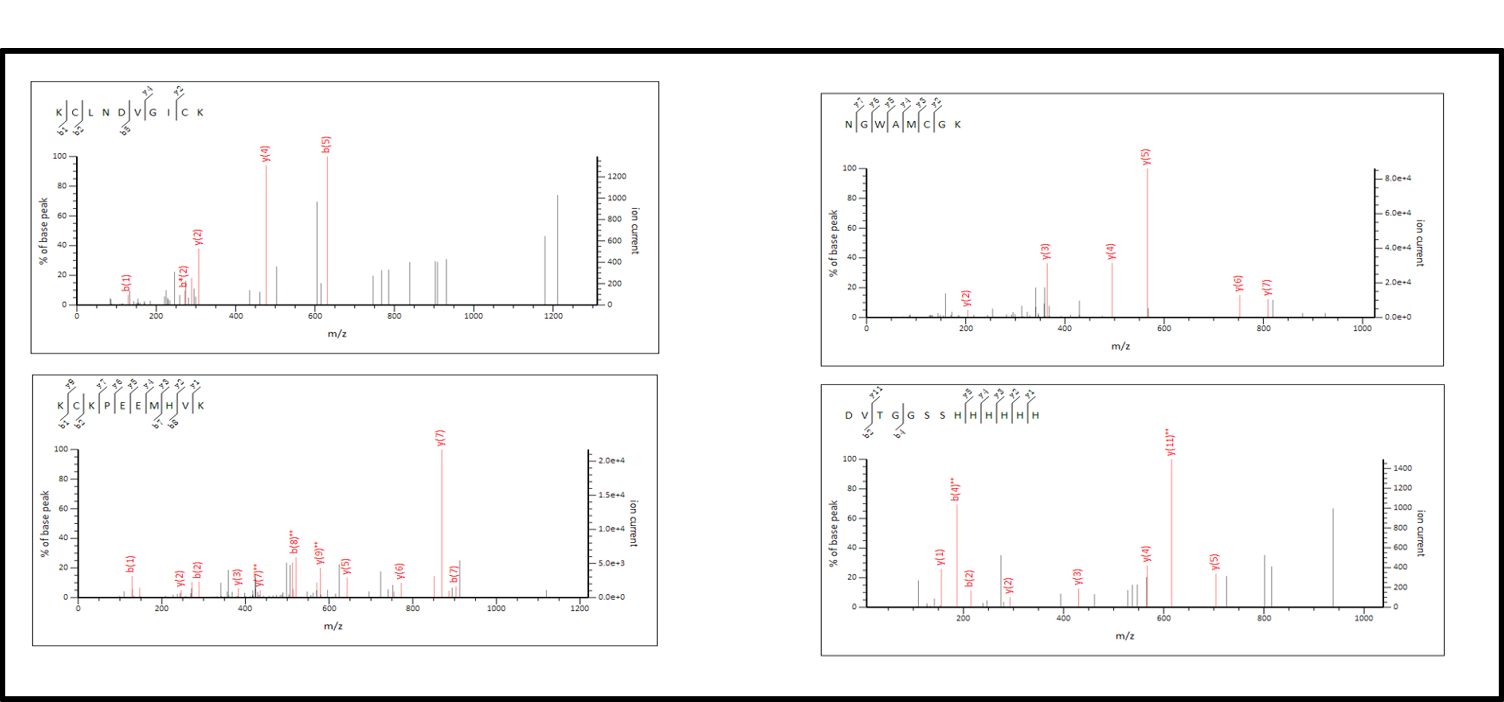

Supplement: Supplementary Figure 4 — Liquid Chromatography-Mass Spectrometry (LC-MS) analysis of recombinant purified hybrid DEFB-TP5 peptide. [file Image_4.tif]
